# The Heterogeneity of Inflammatory Response and Emphysema in Chronic Obstructive Pulmonary Disease

**DOI:** 10.3389/fphys.2021.783396

**Published:** 2021-12-07

**Authors:** Xia Xu, Ke Huang, Fen Dong, Shiwei Qumu, Qichao Zhao, Hongtao Niu, Xiaoxia Ren, Xiaoying Gu, Tao Yu, Lin Pan, Ting Yang, Chen Wang

**Affiliations:** ^1^Department of Immunology, School of Basic Medical Sciences, Capital Medical University, Beijing, China; ^2^Department of Pulmonary and Critical Care Medicine, China-Japan Friendship Hospital, Beijing, China; ^3^Institute of Clinical Medical Sciences, China-Japan Friendship Hospital, Beijing, China

**Keywords:** heterogeneity, eosinophils, emphysema, chronic obstructive pulmonary disease, inflammation

## Abstract

Chronic obstructive pulmonary disease (COPD) is a heterogeneous disease characterized by chronic inflammation, emphysema, airway remodeling, and altered lung function. Despite the canonical classification of COPD as a neutrophilic disease, blood and airway eosinophilia are found in COPD patients. Identifying the tools to assess eosinophilic airway inflammation in COPD models during stable disease and exacerbations will enable the development of novel anti-eosinophilic treatments. We developed different animal models to mimic the pathological features of COPD. Our results show that eosinophils accumulated in the lungs of pancreatic porcine elastase-treated mice, with emphysema arising from the alveolar septa. A lipopolysaccharide challenge significantly increased IL-17 levels and induced a swift change from a type-2 response to an IL-17-driven inflammatory response. However, lipopolysaccharides can exacerbate cigarette smoking-induced airway inflammation dominated by neutrophil infiltration and airway remodeling in COPD models. Our results suggest that eosinophils may be associated with emphysema arising from the alveolar septa, which may be different from the small airway disease-associated emphysema that is dominated by neutrophilic inflammation in cigarette smoke-induced models. The characterization of heterogeneity seen in the COPD-associated inflammatory signature could pave the way for personalized medicine to identify new and effective therapeutic approaches for COPD.

## Introduction

Chronic obstructive pulmonary disease (COPD), a prevalent disease and the third leading cause of death worldwide (GBD 2013 Mortality and Causes of Death Collaborators, 2015), is a heterogeneous disease that consists of several pathological features, including emphysema, chronic bronchitis, small airway remodeling, and vascular remodeling. Characterization of these phenotypes can reflect the complexity of the underlying mechanisms of COPD in individual patients, which may help to identify new and effective therapeutic approaches for COPD.

Mechanistic studies in different animal models have facilitated the discovery of new treatable traits and targets. Several animal models of COPD have been proposed (Wright et al., 2008). Chronic exposure to cigarette smoke (CS) in rodents mimics some features of COPD, including airway inflammation and physiological alterations similar to those in humans. However, the CS model typically requires 3–6 months of exposure, and the features do not progress after the cessation of cigarette smoke exposure (Wright and Churg, 1990). The generally accepted protease-antiprotease hypothesis led to the development of the elastase-induced lung emphysema model, which has a rapid onset and is characterized by prominent air space enlargement without significant small airway inflammation and remodeling. Lipopolysaccharides (LPS) are also used to model COPD because of their proinflammatory effects (Kobayashi et al., 2013), and they usually help to model the exacerbation of COPD (Sajjan et al., 2009). Other proposed models, such as the starvation-induced and apoptosis models, also represent some specific phenotypes. Compared with the pathophysiology of asthma, less is known about the role of eosinophils in the pathophysiology of COPD, despite the finding that up to 40% of patients with COPD have eosinophilic airway inflammation with a similar pattern of expression of type-2 mediators in the airways (George and Brightling, 2016). In this study, we observed that eosinophilic inflammation in the lungs was associated with pancreatic porcine elastase (PPE)-induced emphysema but not with CS-induced emphysema. However, the precise mechanisms by which eosinophils are involved in the development of COPD remain unclear. Addressing this challenge will enable us to use eosinophils as biomarkers to guide the precise management of patients with COPD.

## Materials and Methods

### Animals (COPD Models)

For elastase models, C57BL/6 mice (Charles River) were randomly assigned to four groups: control, emphysema (PPE), LPS, and LPS emphysema groups. Mice in the LPS, PPE, and LPS emphysema groups were induced according to a previously described protocol (Sajjan et al., 2009). Briefly, the mice received four intratracheal instillations of PPE (Sigma Chemical Co., St. Louis, MO, United States) at a concentration of 1.2 U PPE/animal, dissolved in 100 μl of PBS. The control group received sterile PBS (100 μl) using the same protocol. The LPS group received *Escherichia coli* LPS [7 μg LPS from *E. coli* O26:B6 (Sigma-Aldrich, St. Gallen, Switzerland) in 100 μl of PBS] intratracheally, and the fourth group was administered PPE combined with LPS. All mice were anesthetized with 1.5–2.0% isoflurane by mask before each intratracheal instillation, and they were sacrificed 5 or 17weeks after the last instillation.

For CS models, female C57BL/6 mice (Charles River) were exposed to cigarette smoke using a whole-body exposure system (Buxco, Wilmington, NC, United States) as described previously (Pennock et al., 1979). Briefly, the mice were exposed twice daily to six cigarettes for 5 days per week (Monday–Friday), whereas control mice were exposed to room air. All mice were exposed to either CS or room air for 4 months, and were administered LPS (750 ng/kg dissolved in 50 μl sterile PBS) or PBS (50 μl) intratracheally every 2weeks. The mice were euthanized 1week after the last instillation.

### Airway Hyperresponsiveness

One week after the final challenge, airway hyperresponsiveness (AHR) was measured in mice, using a FinePointe noninvasive airway mechanics (NAM) system (Buxco Electronics, Wilmington, NC). Briefly, mice were placed in a double-chamber plethysmograph, which consists of a nasal (head) chamber and a thoracic (body) chamber. Groups of mice were exposed to nebulized DPBS for 5 min. Lung function was recorded and calculated using the FinePointe software to measure expiratory flow at the point where 50% of TV is expired (EF50, ml/s), functional residual capacity (Frc, ml), and specific airway resistance (sRaw, cmH_2_O.S).

### Patients and Lung Samples

Data were also collected from one deceased lung donor and three COPD patients who underwent lung transplantation. All human studies were approved by the Ethical Committee on Human Research of the China-Japan Friendship Hospital (No. 2019-106-K74). Written informed consent was obtained from all patients.

### Cytokine Detection

Mouse sera and mouse lung homogenates were collected. The concentrations of TNF-α, IL-6, IL-1 β, IL12p40, IL-4, IL-5, and IgE levels were measured using SET-Ready-GO ELISA kits (eBioscience) according to the manufacturer’s protocol.

### Flow Cytometry

Anti-mouse antibodies to CD11b, CD11c, Ly6G, and SiglecF were obtained from BD Biosciences. Mouse lungs were perfused and digested into single-cell suspensions, as described previously (Xu et al., 2009). After RBC lysis buffer treatment, the whole lung cells were washed with PBS and stained with the corresponding fluorescent antibodies. Data were analyzed using FlowJo (Tree Star).

### Quantitative PCR

Total RNA was isolated using TRIzol (Invitrogen) and 1 μg of RNA was reverse transcribed using PrimeScript™ RT Master Mix (Takara, RR036A). qPCR was performed using Power SYBR Green PCR Master Mix (TaKaRa, RR420A). The amounts of transcripts were normalized to those of GAPDH.

### Histopathology

For histopathological analyses, the lungs were fixed in a 4% PFA solution, processed, and embedded in paraffin according to standard procedures. Next, 5- μm sections were stained with hematoxylin and eosin (H&E), and the stained sections were scanned using a Nikon microscope. The mean linear intercept (×200 magnification) in the pulmonary tissue was determined by the point counting technique across 10–20 random, non-overlapping microscopic fields.

### Statistical Analysis

For animal experimental data, all values in this study are presented as mean ± SEM, unless stated otherwise. All experiments were replicated at least three times, and statistical significance was calculated by a two-tailed t-test, using GraphPad Prism software. Statistical significance was set based on values of *p*: ns, *p* > 0.05; *p < 0.05, **p < 0.01, ***p < 0.001.

## Results

### Murine Model of Chronic Pulmonary Inflammation Mimics Pathological Features of COPD

**Figure 1:**
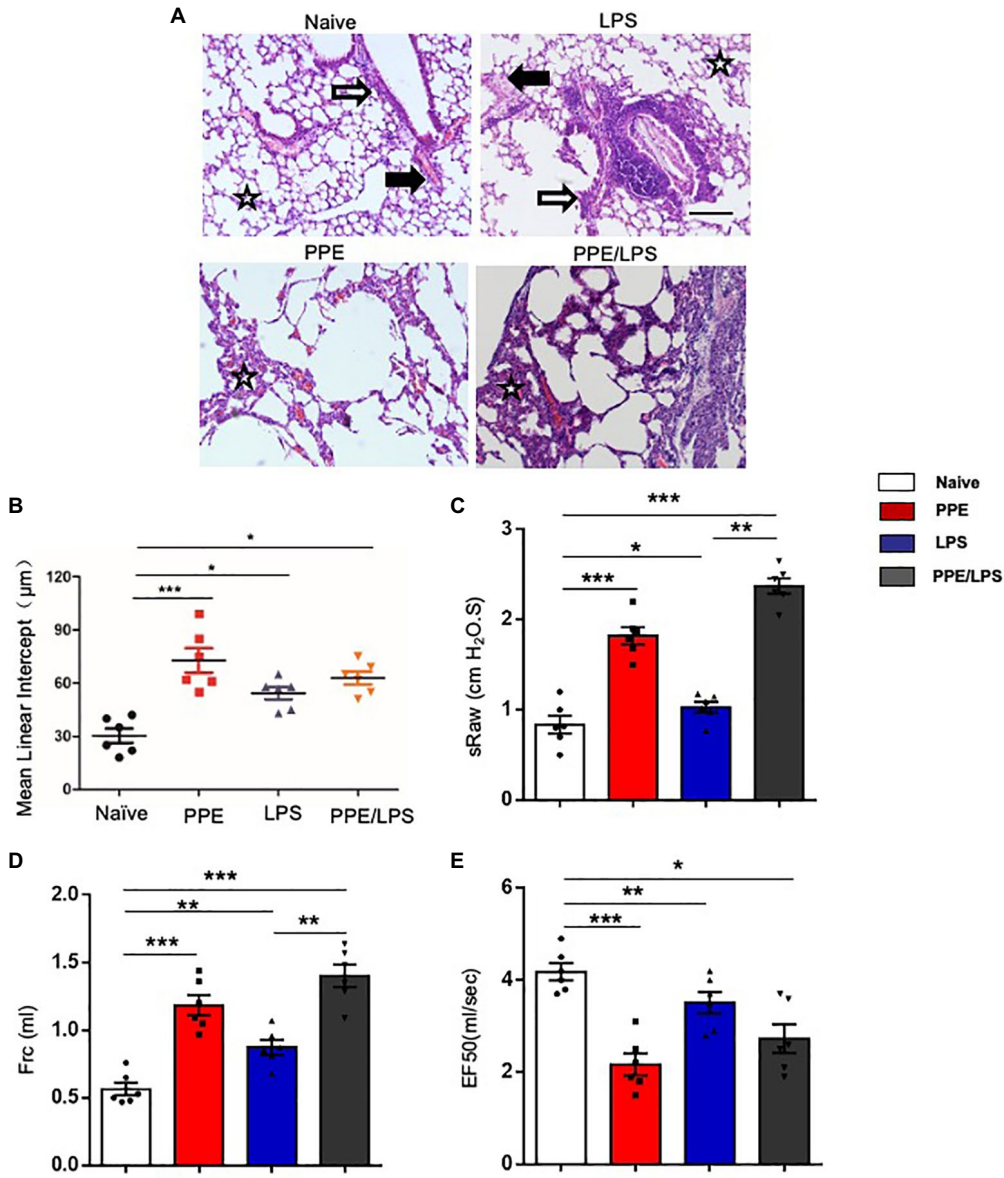
Emphysema induced by multiple elastase instillations. **(A)** C57BL/6 mice were intranasally administered LPS (7 mg), elastase (1.5 U), or a 100 ml combination of LPS and elastase once a week for 4weeks. A terminal analysis was performed 1week after the last challenge. Pulmonary pathological sections were stained with hematoxylin/eosin (HE), and visualized at the indicated magnifications. **(B)** Mean linear intercept was measured in the lungs of treated mice. (×200), Bar: 100 μm. **(C–E)** Lung function was measured using the FinePointe™ NAM system. sRaw, specific airway resistance; Frc, functional residual capacity; EF50, expiratory flow at the point where 50% of TV is expired. Data are presented as mean ± SEM. *p < 0.05, **p < 0.01, ***p < 0.001; two-tailed unpaired t-test. Hollow arrow: airway. Solid arrow: vessels. Star: interstitia and alveoli.

Human COPD is a heterogeneous disease characterized by chronic inflammation, emphysema, airway remodeling, and altered lung function. We aimed to develop different animal models of COPD that accurately recapitulate the complex multifaceted features of this disease (Supplementary Figure 1A). PPE mainly models the emphysema feature of COPD, and LPS helps to model the exacerbation of COPD. Intratracheal instillation of PPE induced severe emphysema in the lungs of the treated mice (Figures 1A,B). LPS instillation induced marked inflammation, and inflammatory cells were observed around the airways and large vessels (Figure 1A). Next, we assessed pulmonary function using the FinePointe NAM system and found that mice that were sensitized and challenged with PPE or LPS for 5weeks had a significant enhancement in airway obstruction compared with PBS control mice. In addition, sRaw and Frc values increased in both PPE- and LPS-treated mice (Figures 1C,D), whereas EF50 values decreased significantly upon PPE or LPS treatment (Figure 1E).

### Eosinophils Accumulated in PPE-Induced Emphysema Models

**Figure 2:**
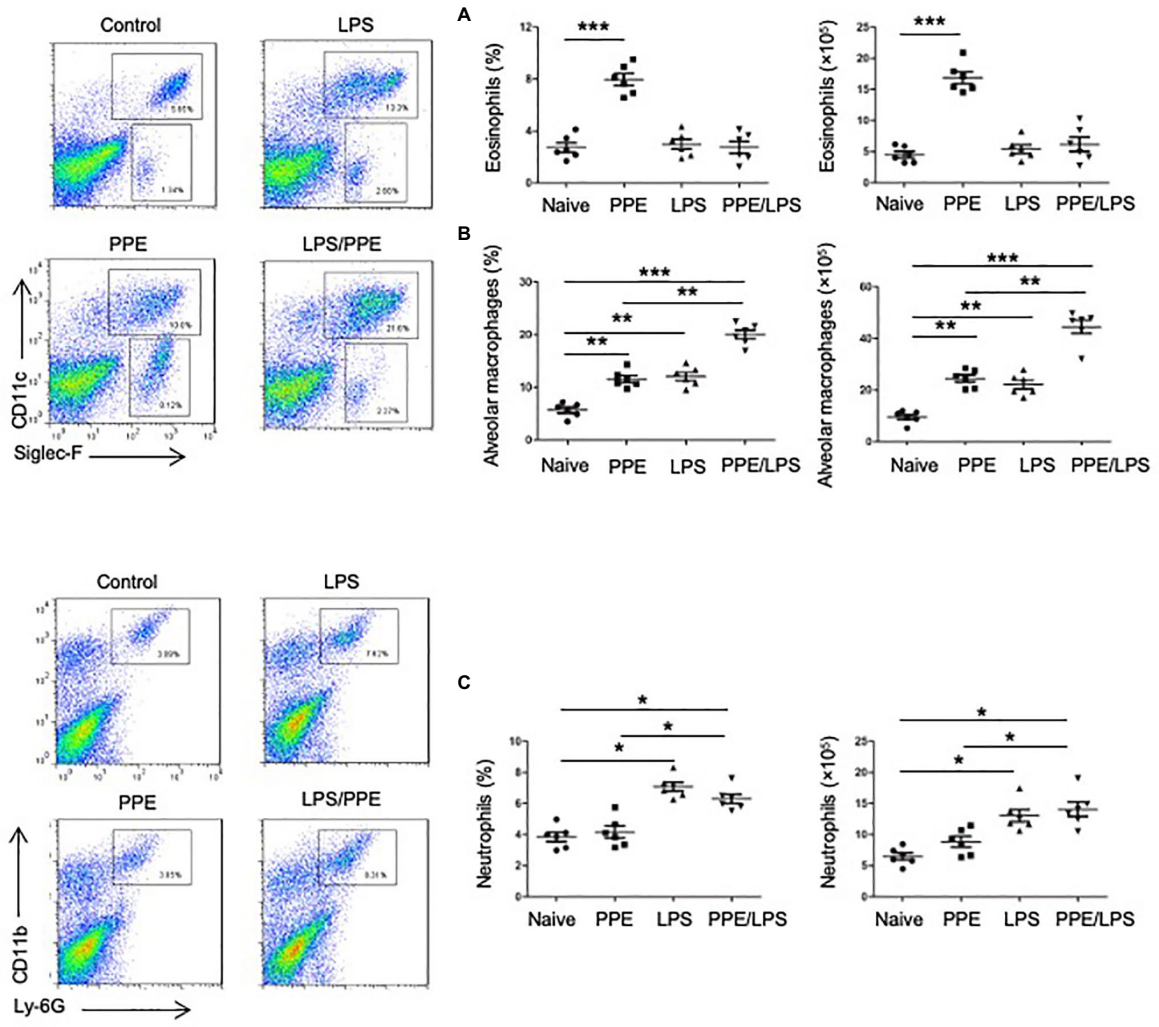
Eosinophils accumulated in PPE-induced emphysema models. **(A)** Eosinophil (SiglecF^+^) and neutrophil (Ly6G^+^) counts in lungs of mice used for the indicated treatment. **(B,C)** Total lung cells were isolated and stained with a live/dead staining kit, followed by staining with fluorochrome-conjugated Abs corresponding to the various cell populations. Data shown are representative of three independent experiments. Data are presented as mean ± SEM. ^*^p < 0.05, ^**^p < 0.01, and ^***^p < 0.001; two-tailed unpaired t-test.

We determined the cellular composition of the lungs by flow cytometry (Supplementary Figures 2A,B) and found that mice challenged with PPE showed a prominent eosinophil-biased response (Figure 2A), whereas mice exhibited greater neutrophil accumulation in the lungs after LPS instillation or PPE combined with LPS challenge (Figure 2C). LPS instillation also exacerbated alveolar macrophage accumulation in the PPE- combined with LPS-challenged mice (Figure 2B).

### Type-2 Response Elicited in Elastase-Treated Mice

**Figure 3:**
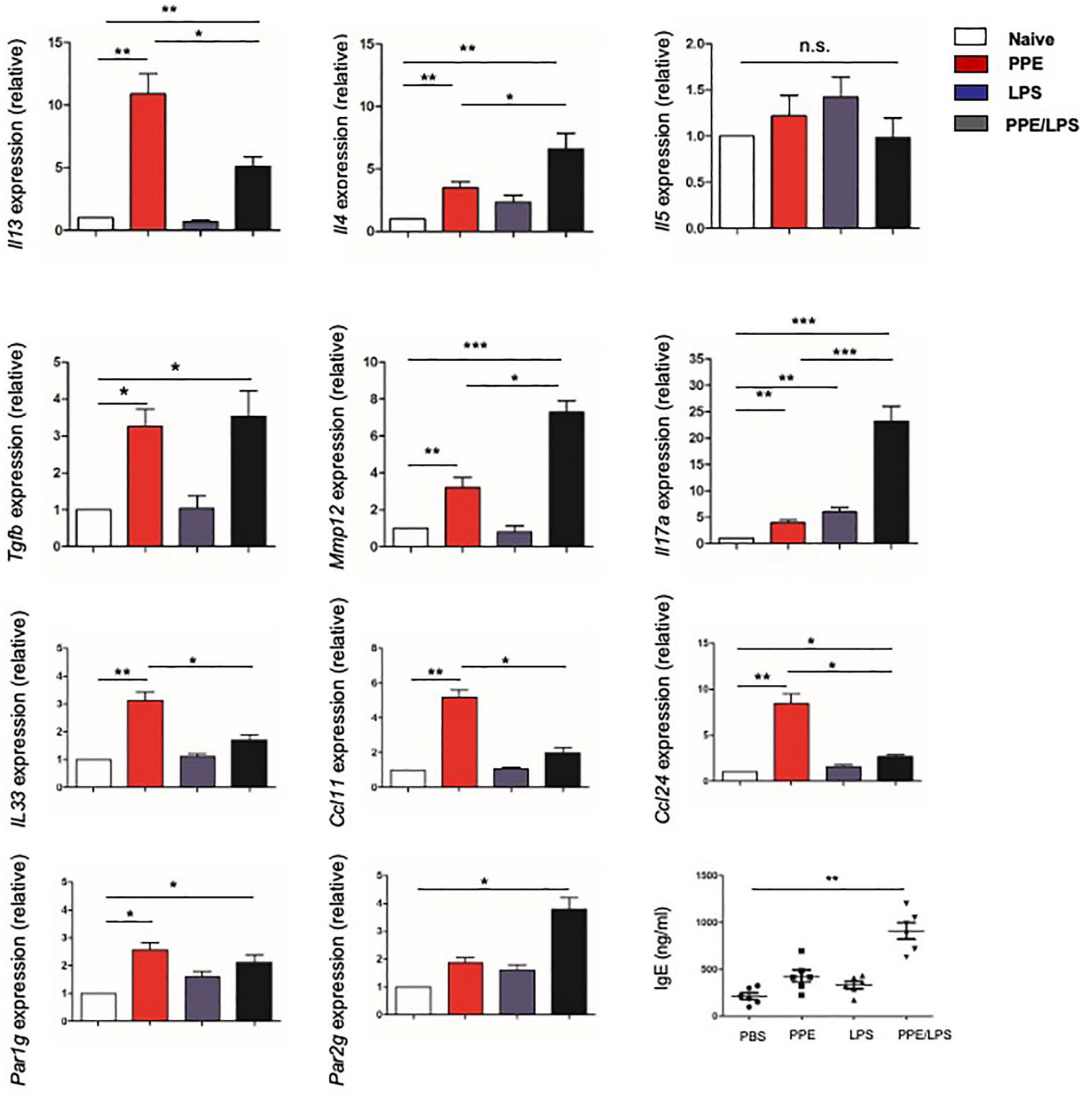
Type-2 response elicited in elastase-treated mice. The indicated gene expression in lungs from mice treated with LPS or elastase was quantified by real-time PCR and normalized to GAPDH. IgE levels in sera were measured by ELISA. Data are presented as mean ± SEM. ^*^p < 0.05, ^**^p < 0.01, and ^***^p < 0.001; two-tailed unpaired t-test.

Furthermore, we assessed the chronicity of inflammatory lung diseases by measuring the expression of Th1 (IFN-γ) and Th2 (IL-4, IL-5, and IL-13) cytokines in the lungs of treated mice and found that lung cells from PPE-treated mice expressed elevated levels of IL-4, IL-5, and IL-13 (Figure 3), but not IFN-γ (data not shown). However, elastase-pulsed LPS treatment, but not LPS alone, enhanced MMP12 and IL-17A production (Figure 3). While protease activated receptors (PARs) are activated by many proteases and are capable of inducing a wide range of inflammatory processes and cytokines within the lung, we assessed the expression of PARs. The results showed that lung cells from PPE-treated mice expressed elevated levels of PAR-1; however, elastase-pulsed LPS treatment enhanced PAR-2 expression. In addition, we detected the expression of eosinophil chemoattractants and alarmins such as IL-33. The expression of CCL11, CCL24, and IL33 increased significantly in mice treated with PPE alone (Figure 3), which may contribute to PPE-induced eosinophilia in the lung. These results suggest that Th2 inflammation exists in a COPD subset, but neutrophilic inflammation is enhanced after LPS treatment. Further measurement of serum IgE showed increased levels in LPS/elastase-treated mice (Figure 3).

### Neutrophilic Inflammation in CS-Induced Chronic Experimental COPD Models

**Figure 4:**
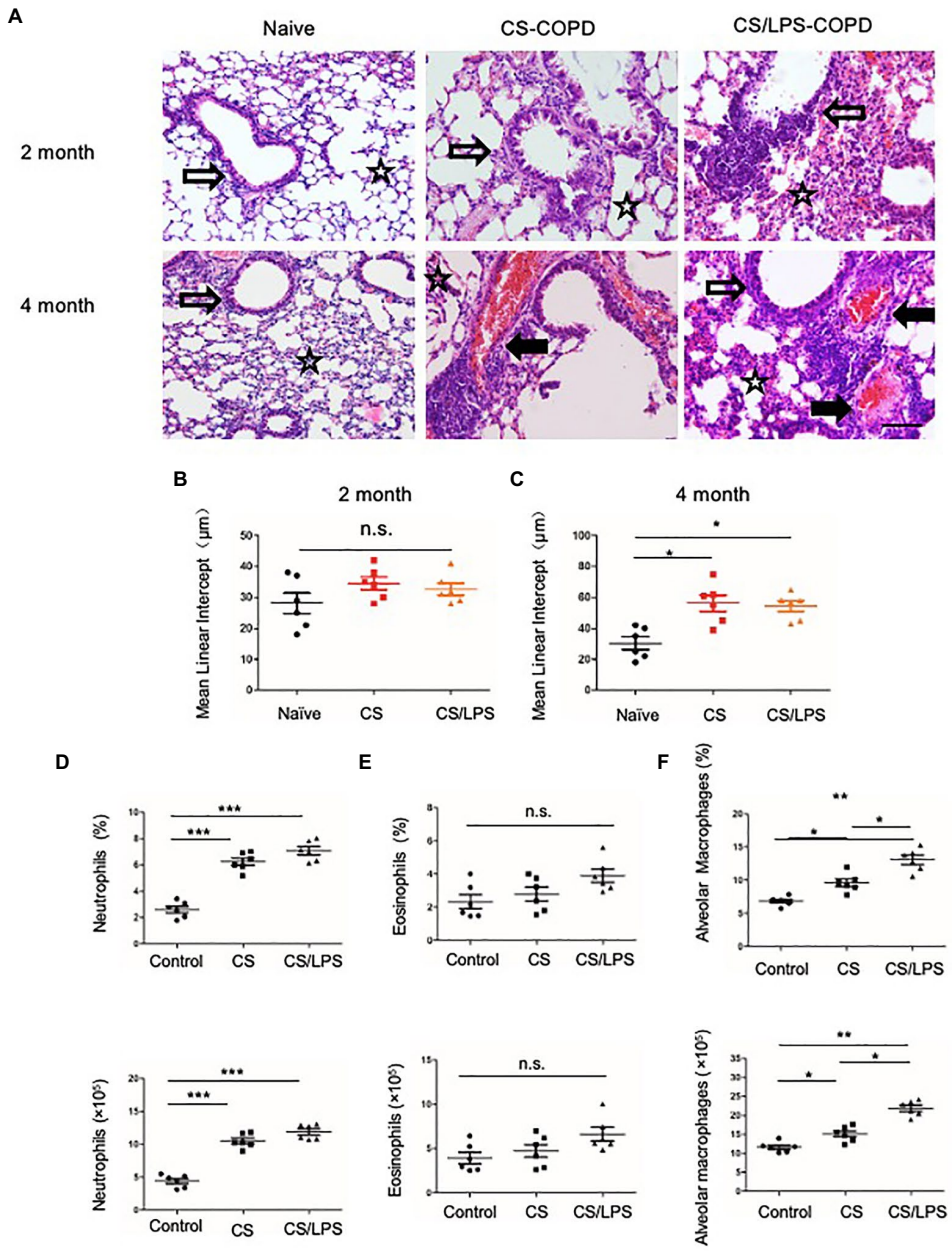
The association between emphysema and eosinophils in CS-induced COPD models. C57BL/6 mice were exposed to CS or CS combined with intratracheal instillation of LPS for 2 or 4 months. **(A-C)** The mean linear intercept was measured in the lungs of the treated mice after exposure to CS or CS combined with LPS compared to that measured in the control groups at 2 or 4 months; (×200), Bar: 100 μm. **(D-F)** Total lung cells were isolated from mice in the chronic experimental COPD groups after 4 months of treatment. Neutrophil (CD11b^+^Ly6G^+^), eosinophil (SiglecF^+^) and alveolar macrophage (CD11c^+^ SiglecF^+^) counts were analyzed by FACS. *p < 0.05, **p < 0.01 and ***p < 0.001; two-tailed unpaired t-test. Hollow arrow: airway. Solid arrow: vessels. Star: interstitia and alveoli.

Smoking is a major risk factor for COPD. CS-induced chronic experimental COPD models were generated using a whole-body smoke exposure system. (Figure 4A; Supplementary Figure 1B). Mice that were sensitized and challenged with CS and LPS exhibited an enhanced AHR for 2 months compared with those in the PBS control group (Supplementary Figure 3A). However, no significant increase in emphysema was observed in CS-exposed groups compared with the control group exposed to room air (Figure 4B) until after 4 months of treatment when there was a modest increase in emphysema (*p* < 0.05) in the lung parenchyma of mice in the CS-exposed groups compared with that of mice in the control group (Figure 4C). We observed no significant increase in eosinophils in the CS-exposed or CS/LPS groups compared with those in the control group during the 4-month treatment (Figure 4E; Supplementary Figure 4). The results showed increased densities of alveolar macrophages and neutrophils in the lung parenchyma in the CS and CS/LPS groups compared with those in the control group, especially for groups compared with CS groups with CS/LPS groups (Figures 4D,F; Supplementary Figure 4), suggesting that LPS exacerbates the neutrophilic inflammatory response in CS-induced inflammation. However, we found that there was a sustained significant increase in eosinophils in PPE-treated mice after 4 months (Supplementary Figure 3B).

### The Collagen Distribution in Experimental COPD Models

**Figure 5:**
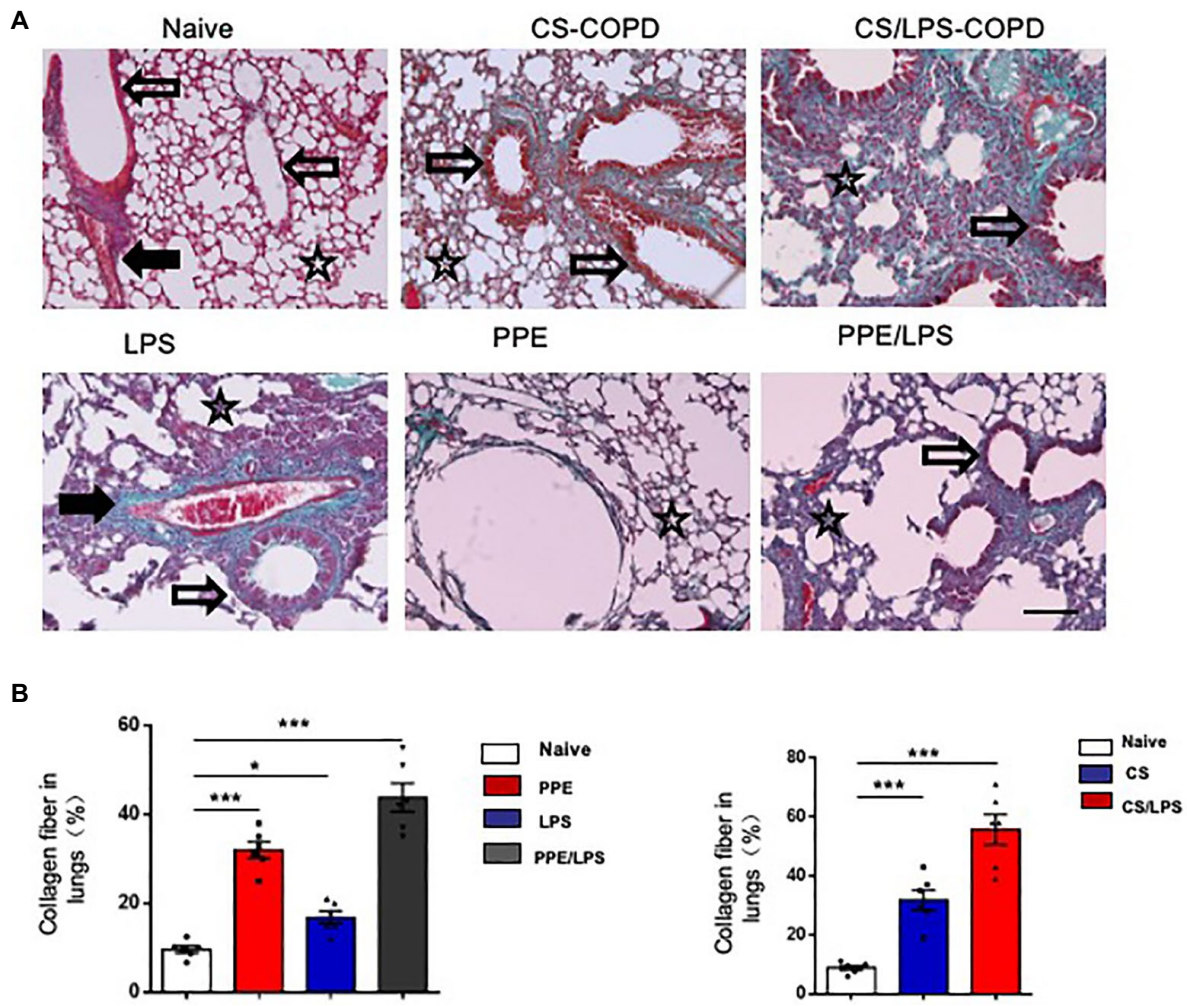
Collagen distribution in experimental COPD models. C57BL/6 mice were assigned into two groups. One group was treated with intratracheal instillation of elastase or PBS for 2 or 4 months, while the other group was exposed to CS or room air for the indicated time. Collagen fiber content and representative photomicrographs of **(A)** alveolar septa and **(B)** airways of animals after the indicated treatment, stained with Masson’s trichrome; (×200), Bar: 100 μm. Data are presented as mean ± SEM; *p < 0.05, **p < 0.01, and ***p < 0.001; two-tailed unpaired t-test. Hollow arrow: airway. Solid arrow: vessels. Star: interstitia and alveoli.

Small airway remodeling is well-recognized as an important cause of airflow obstruction in patients with COPD. Most of the increased wall areas were composed of collagen and fibronectin. In the PPE-treated groups, we observed a slight increase in collagen fiber deposition, mainly in the lung alveolar septa, for 2 months. After treatment for 4 months, the amount of collagen fibers increased and was distributed around the pulmonary airways and vessel walls. These results suggest that emphysema exacerbation results in continuously increased alveolar destruction and airspace heterogeneity in PPE-primed lungs (Figures 5A,B). In contrast, for the first 2 months, the increased collagen deposition was mainly around the airways and pulmonary vessel walls in the CS-exposed groups. The amount of collagen fibers increased in the peribronchial airspaces and alveolar septa for 4 months, suggesting that inflammation of the small airways and destruction of bronchiolar-alveolar attachments contribute to the development of emphysema (Figures 5A,B).

### The Heterogenicity of COPD Patients

**Figure 6:**
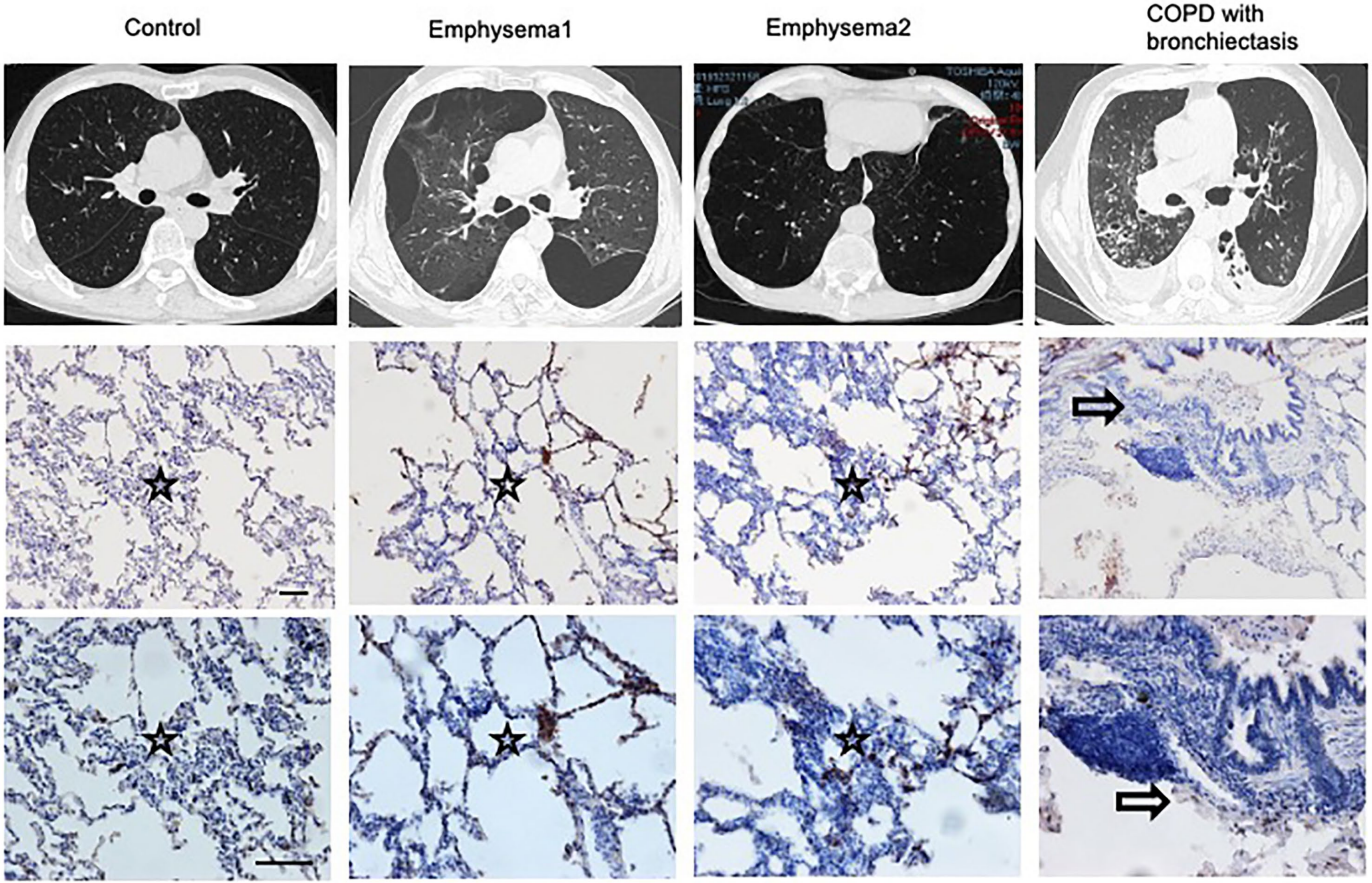
Heterogenicity of COPD patients. CT scans and lung slices from a donor (Control), two patients with emphysema, and one COPD patient with bronchiectasis who received lung transplantation. (Upper, × 100; Bottom, × 200), Bar: 100 μm. Hollow arrow: airway. Star: interstitia and alveoli.

The phenotypes of COPD are heterogeneous. We compared the CT scans and lung slices of human lungs and observed that patients with emphysema had higher blood eosinophil levels, more prominent lung eosinophil infiltration, and a Th2 reaction. A patient with bronchiectasis had prominent airway inflammation, lower blood eosinophil levels, and fewer lung eosinophil infiltrations (Figure 6; Supplementary Table 1).

## Discussion

Emphysema and chronic bronchitis are the most important conditions that compose COPD, which is characterized by different physiopathologies and symptoms (Brightling and Greening, 2019). T1 and T17 immunity are the most common phenotypes with eosinophil-associated T2-mediated immunity, and autoimmunity is observed in more severe diseases (Yun et al., 2018). Mature circulating blood eosinophils are recruited to the airways by immunoregulatory cells and chemokines. Eosinophils have been reported to have an immunomodulatory role in airway diseases. They synthesize and secrete proinflammatory cytokines, chemokines, and growth factors, resulting in eosinophilic inflammation. The blood eosinophil count has been proposed as a biomarker for directed therapy in COPD. Clinical studies evaluating both mepolizumab and benralizumab in eosinophilic COPD patients, the sub-population of COPD patients most likely to respond to anti-eosinophil therapy, remain unclear (Bel and Ten Brinke, 2017; Pavord et al., 2017; Criner et al., 2019). Further long-term trials focused on the effect of anti-eosinophil treatment for the development of emphysema in COPD patients with eosinophilic phenotype are warranted.

α1-Antitrypsin is a neutrophil elastase inhibitor, and its deficiency is associated with a poorly-controlled release of elastase, resulting in the destruction of elastin in the lung tissue, a process mainly associated with the development of emphysema (Stockley and Turner, 2014). Developing an elastase-induced emphysema model is an interesting, low-cost approach that demonstrates histological and morphological characteristics compatible with those of panacinar emphysema. Elastolytic enzymes may augment inflammatory cell influx into the airspaces, which in turn promotes the release of matrix metalloproteases (MMPs) and reactive oxygen species. The matrix-degrading capacity of MMP causes destruction of the alveolar septa, increases airspaces, and causes pulmonary and cardiovascular dysfunction. Elastin fragments resulting from alveolar destruction become chemoattractants for further inflammatory cell influx (Wierzchon et al., 2017). Recent data from animal and human studies suggest that the pathophysiology of elastase emphysema differs from that of smoke-induced emphysema. In smoke-exposed mouse lungs, an increase in apoptotic cells has also been described, and may be associated not only with the extrinsic pathway and an enhanced expression of FasL and caspase but also with mitochondrial pathway activation. Sawada et al. demonstrated that apoptosis in elastase-induced emphysema is not mediated by the Fas/FasL interaction. However, the extrinsic pathway can also be activated by other members of the TNF superfamily, including the TNF receptor, TRAIL-1/TRAIL-2, and lymphotoxin β receptor (Rangasamy et al., 2009). These data support the idea that the pathophysiological specificities of each model require further investigation.

In our mouse model, we found that lung eosinophilia was associated with airspace enlargement and emphysema. However, the mechanism and function of eosinophils remain unclear. It has been suggested that eosinophil-derived IL-13 enhances emphysema by stimulating macrophages to secrete MMP-12 (Shibata et al., 2018). However, in asthma, despite the higher frequency of eosinophilic inflammation, airspace enlargement is more likely to be a sign of trapped air following airway remodeling than emphysema (Gelb et al., 2018). This suggests that additional mechanisms of eosinophilic inflammation are required to induce alveolar wall destruction. The pathophysiological specificities of each model require further investigation.

In addition, we found that eosinophils associated with emphysema arose from the alveolar septa, thereby distinguishing it from the small airway disease-associated emphysema in CS-induced models. In this regard, focus should be on the aspects of disease that are independent of eosinophilic inflammation and those where the eosinophils play a key role. It is also necessary to understand the relative merits of each therapy in an individual and application of biomarkers to predict long-term outcomes.

## Data Availability Statement

The original contributions presented in the study are included in the article/Supplementary Material, further inquiries can be directed to the corresponding authors.

## Ethics Statement

The studies involving human participants were reviewed and approved by the Ethical Committee of the China-Japan Friendship Hospital. The patients/participants provided their written informed consent to participate in this study. The animal study was reviewed and approved by the Ethical Committee of the China-Japan Friendship Hospital.

## Author Contributions

XX, KH, QZ, and LP performed the experiments. TY, HN, and XR collected patient samples. FD and XG performed data analysis. XX, KH, and SQ wrote the paper. TY and CW designed the study. All authors contributed to the article and approved the submitted version.

## Funding

This work was supported by the Natural Science Foundation of Beijing (7202130 to XX), the National Natural Science Foundation of China (L1422025 to TY), and the CAMS Innovation Fund for Medical Sciences (2018-I2M-1-001 to CW).

## Conflict of Interest

The authors declare that the research was conducted in the absence of any commercial or financial relationships that could be construed as a potential conflict of interest.

## Publisher’s Note

All claims expressed in this article are solely those of the authors and do not necessarily represent those of their affiliated organizations, or those of the publisher, the editors and the reviewers. Any product that may be evaluated in this article, or claim that may be made by its manufacturer, is not guaranteed or endorsed by the publisher.
