# Supplementary material for: The Heterogeneity of Inflammatory Response and Emphysema in Chronic Obstructive Pulmonary Disease
Source: Front Physiol. 2021 Dec 7;12:783396. doi: 10.3389/fphys.2021.783396 (PMC8689000; doi:10.3389/fphys.2021.783396)
Supplement: Supplementary file 1 [file Data_Sheet_1.docx]

Supplementary Material

# Supplementary Figures and Tables

## Supplementary Figures

**
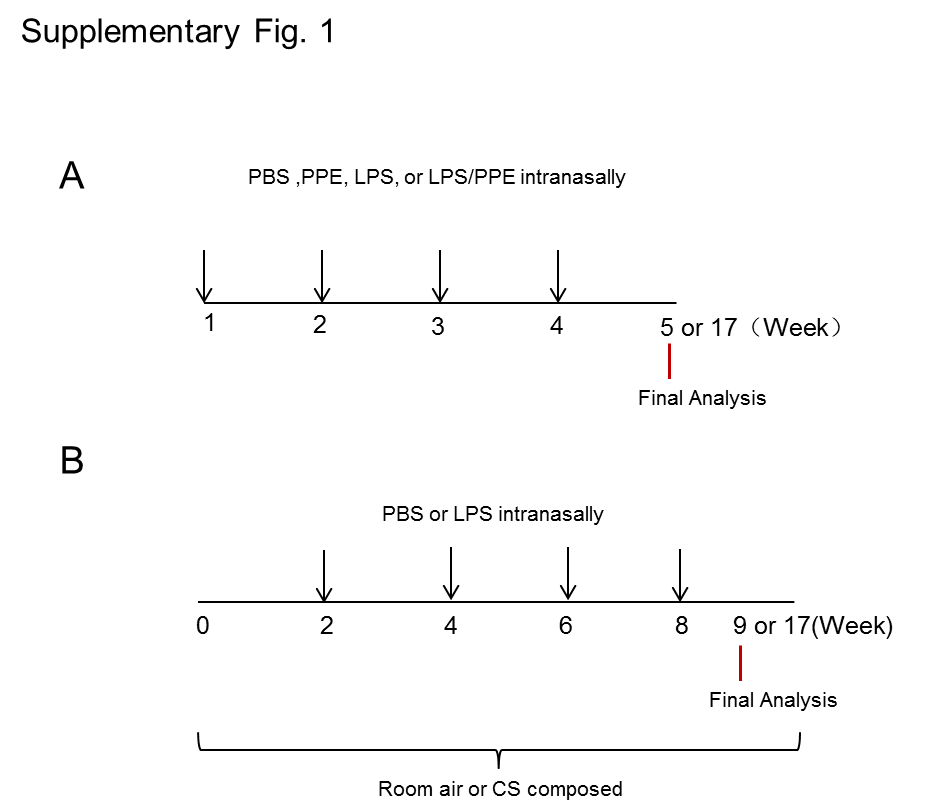
**

**Supplementary Figure 1.** Animal experimental protocol. **(A)** For elastase models, mice were randomly assigned into four groups: Control (PBS), emphysema (PPE), LPS, and PPE/LPS. In each group, animals received four or sixteen intratracheal instillations at 1-week intervals. One week after the last instillation, the mice were sacrificed, and data were analyzed. **(B)** For CS models, the mice were exposed to smoke or room air. From the end of the 2nd week, mice were injected with LPS (750 ng/kg dissolved in 50 μL sterile PBS) or PBS (50 μL) intratracheally every two weeks. One week after the last instillation, mice were sacrificed, and data were analyzed.


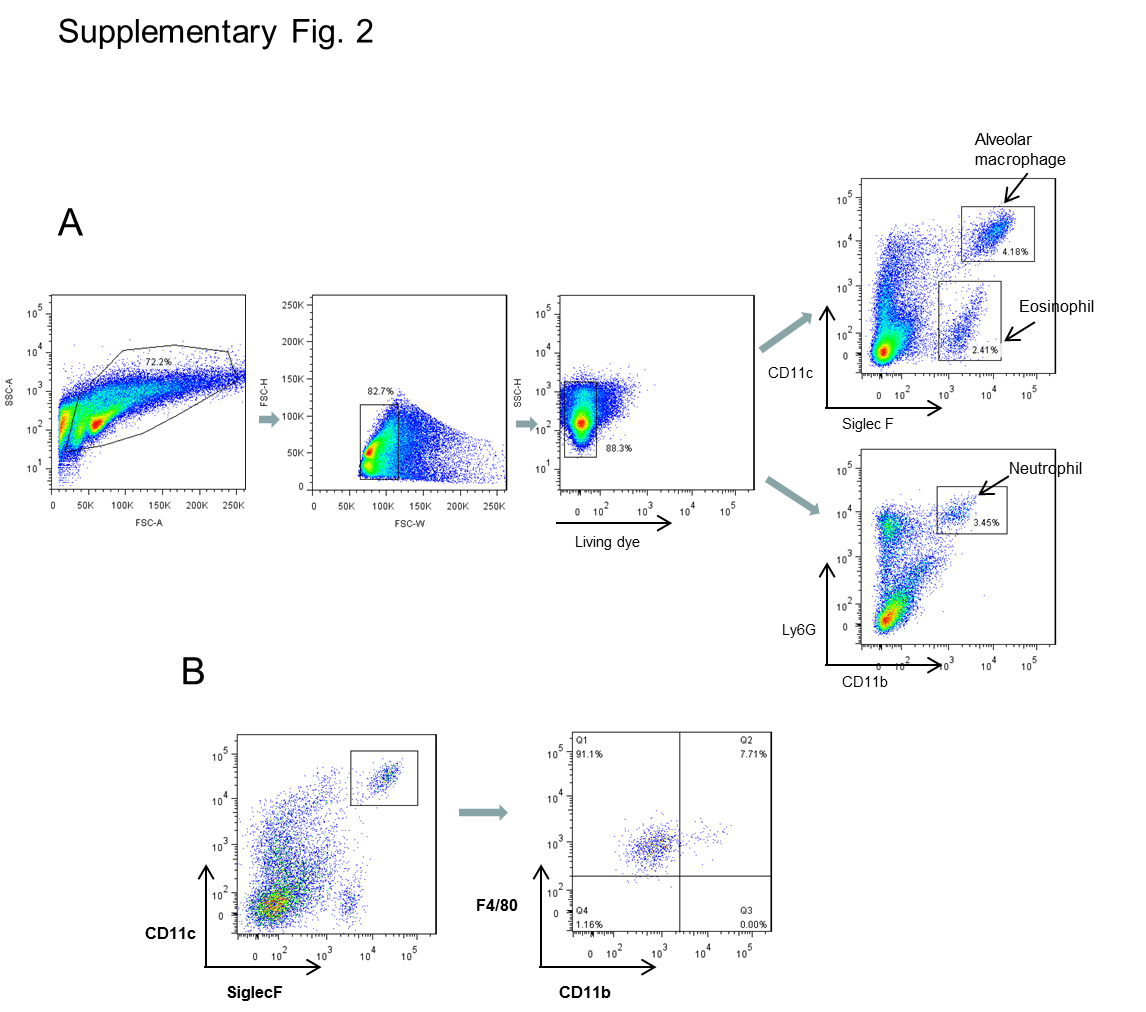


**Supplementary Figure. 2** **(A)** Gating strategy for flow cytometry to analyze alveolar macrophage (CD11c+ SiglecF+), eosinophil (SiglecF+), and neutrophil (Ly6G+) counts in the lungs. **(B)** Raw data of the flow cytometry for alveolar macrophages in the lungs.

**
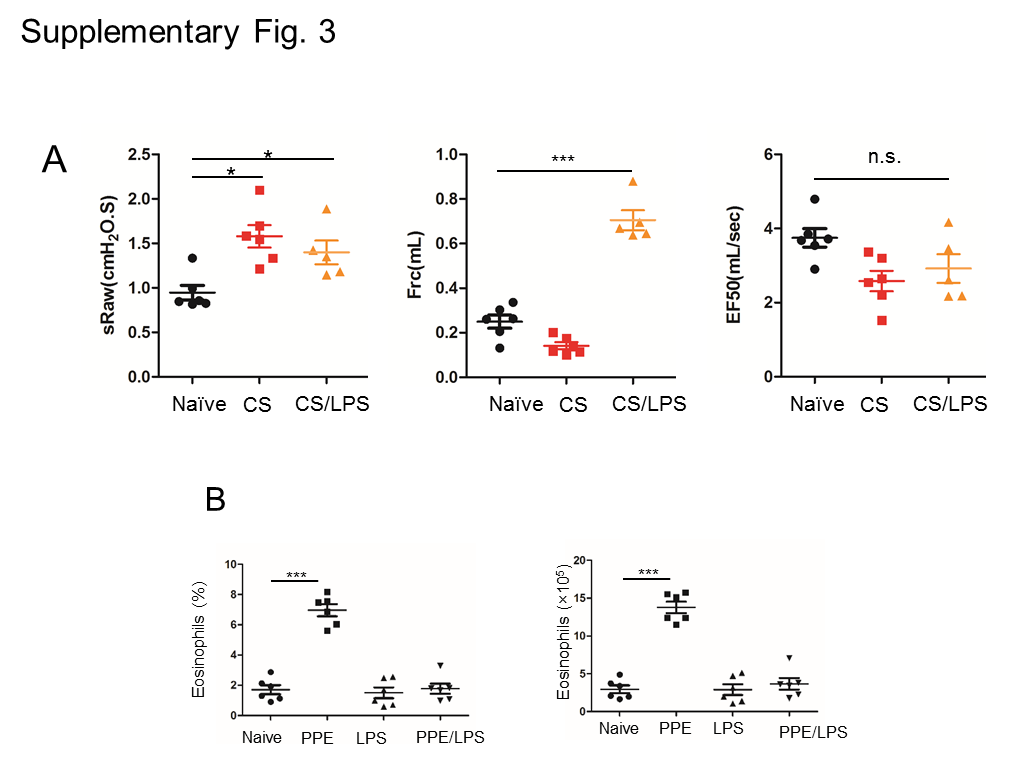
**

**Supplementary Figure 3.** **LPS exacerbates cigarette smoking-induced airway inflammation**

The mice were exposed to the smoke of six cigarettes per day, and on the weekends of the second week, injected with LPS (750 ng/kg dissolved in 50 μL PBS, Sigma-Aldrich) or PBS (50 μL) intratracheally. One week after the last challenge, the mice were sacrificed. Lung tissue sections from C57BL/6 mice were stained with hematoxylin and eosin. Mean linear intercept was measured. **(A)** Lung function was measured using the FinePointe™ NAM system. sRaw: specific airway resistance. Frc: Functional residual capacity EF50: expiratory flow at the point 50% of the TV expires. **(B)** C57BL/6 mice were exposed to CS or CS combined with intratracheal instillation of LPS for 4 months, and total lung cells were isolated from mice in the chronic experimental COPD groups after 4 months of treatment. Eosinophil (SiglecF^+^) counts were analyzed by FACS; data are represented as mean ± SEM. *P < 0.05, **P < 0.01, ***P < 0.001, statistically analyzed by two-tailed unpaired t-test.

**
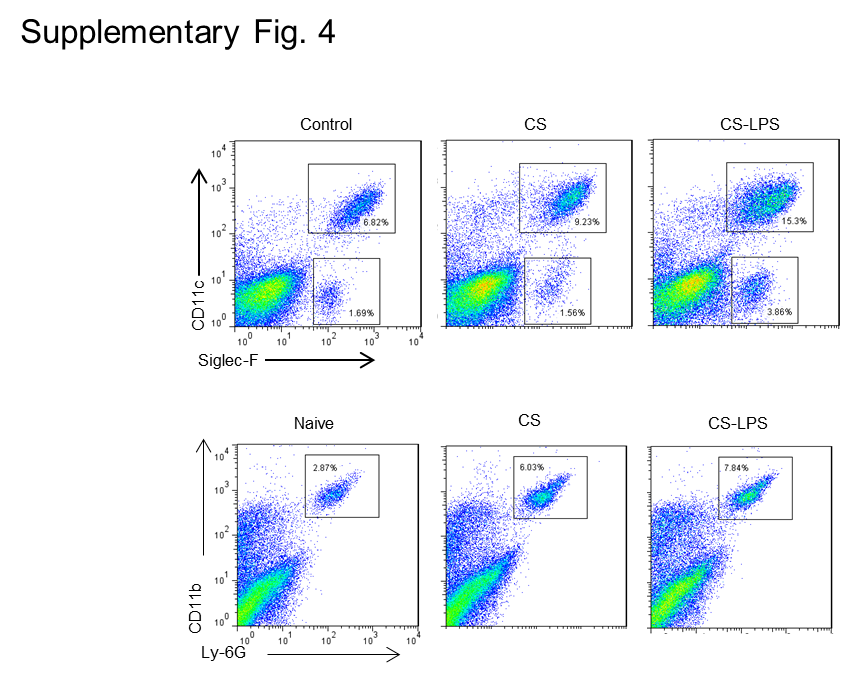
**

**Supplementary Figure 4.** Flow cytometry analysis of lung eosinophil (SiglecF+) and neutrophil (Ly6G+) subsets in total lung cells of mice exposed to CS or CS combined with intratracheal instillation of LPS for four months

## Supplementary Tables

**Supplementary Table 1.**

|  | **Patient 1** | **Patient 2** | **Patient 3** |
| --- | --- | --- | --- |
| Diagnosis | Emphysema | Emphysema | COPD with bronchiectasis |
| Age/sex | 62/male | 51/male | 65/male |
| Blood eosinophils (uL) | 540 | 420 | 130 |
| CD4^+^/CD8^+^ T cell | 2.96 | 0.83 | 1.19 |
| mPAP (mmHg) | 40 | 31 | 55 |
| FEV_1_ (L) | 0.56 | 0.35 | 0.49 |
| FEV_1_% pred (%) | 35.4 | 10.0 | 39.4 |
| FEV_1_/FVC | 45.3 | 29.7 | 39.5 |
| DLCO/VA (%) | 22.8 | intolerable | 32.5 |

Abbreviations: COPD, chronic obstructive pulmonary disease; DLCO/VA, diffusing capacity of the lungs for carbon monoxide (DLCO) divided by the alveolar volume (VA); FEV_1_, forced expiratory volume in one second; FEV_1_% pred, the ratio of measured FEV_1_ over predicted FEV1; FEV1/FVC, the ratio of FEV1 over forced vital capacity (FVC); mPAP, mean pulmonary artery pressure.

**Supplementary Table 2.**

| **Antibody** | **Catalog number** | **Distributor** |
| --- | --- | --- |
| **For Flow cytometry** |  |  |
| PE-anti-mouse SiglecF (E50-2440) | 562068 | BD Pharmingen |
| PerCP-Cy5.5 -anti-mouse CD11b (M1/70) | 561114 | BD Pharmingen |
| APC-anti-mouse CD11c(HL3) | 561119 | BD Pharmingen |
| BV421-anti-human-CD11b ( D12 ) | 742637 | BD Pharmingen |
| APC-eFluor®780-anti-mouseF4/80(BM8) | 47-4801-82 | eBioscience |
| **For IHC** |  |  |
| Rabbit anti-human-PRG2 | Isbio | LS-C806441 |
